# ALSPAC parents’ descriptions of childhood stresses in their parents and grandparents

**DOI:** 10.12688/wellcomeopenres.16732.2

**Published:** 2022-06-14

**Authors:** Karen Birmingham, Yasmin Iles-Caven, Jean Golding

**Affiliations:** 1 Centre for Academic Child Health, Bristol Medical School, University of Bristol, Oakfield House, Oakfield Grove, Bristol, BS8 2BN, UK

**Keywords:** ALSPAC, Family History, Childhood Stress, Transgenerational Inheritance, Grandparents, Great-grandparents

## Abstract

**Background: **There is evidence that childhood stresses or traumas influence individuals’ descendants’ health and wellbeing through epigenetic mechanisms. However, few longitudinal studies have details of such ancestral data.

**Methods: **Nearly 7,000 parents of the original Avon Longitudinal Study of Parents and Children (ALSPAC) cohort completed questionnaires concerning their parents’ and grandparents’ childhoods. As part of a questionnaire validation exercise  we conducted recorded interviews with 100 of these parents. Here we describe some of the vivid accounts from these interviews of stresses encountered by the parents’ ancestors.

**Results: **The interviews provided insights into the childhoods of two previous generations of this cohort, most of whom had lived through one, if not two, World Wars. Many children were brought up, not by their parents but by relatives or acquaintances and/or left home very young to ‘go into service’ or start work. A few interviewees had wealthy relatives with nannies and governesses and attended expensive boarding schools but by far the most frequent accounts were of poverty, often severe, with related lack of education and illiteracy, alcoholism and violence, alongside devastating effects of the World Wars.

**Conclusions: **Although the interviews focussed on stresses in childhood and therefore the accounts seemed somewhat negative, many interviewees described their relatives as having secure, stable childhoods. Of the many struggling families though, the predominant impression was their remarkable resilience; all went on to have children or grandchildren who are stable enough to participate for three decades, entirely altruistically, in ALSPAC.

## Introduction

The Avon Longitudinal Study of Parents and Children (ALSPAC) was established to understand how genetic, biological, environmental, social, psychological and psychosocial factors influence the health and development of children and their parents. ALSPAC is a multi-generation prospective cohort based in Bristol in the South West of England. More than 14,000 pregnant women were enrolled in 1991 and 1992. Their partners, children and now grandchildren have been recruited and followed up over multiple time points. A wide variety of biological samples have been collected along with a vast array of exposure and outcome data collected via questionnaire, face-to-face clinics and through linkage to administrative data. ALSPAC provides a rich resource for many national and international researchers from many disciplines to study the environmental and genetic factors that affect health and development ( ALSPAC website, Boyd *et al.*, 2013; Fraser *et al.*, 2013).

One of many ALSPAC projects involves identifying childhood stresses in the parents and grandparents of the ALSPAC mothers and fathers. There is increasing evidence that these stresses can influence descendants, independently of social patterning, probably through epigenetic mechanisms ( Pembrey *et al.*, 2014; Pembrey, 2018). Nearly 7,000 ALSPAC parents completed questionnaires concerning their own parents’ and grandparents’ childhoods ( Golding *et al.*, 2020). Validation of these questionnaires was necessary. This involved recorded interviews with 100 of the parents who had completed questionnaires. The interviews allowed the parents to talk freely about their ancestors’ childhood in some detail, frequently accompanied by vivid accounts of the stresses encountered by their relatives. Secondary to the validation analyses, the descriptions which emerged from the interviews provided fascinating insights into the childhoods of two previous generations of this cohort. Much of this information, such as sibling deaths or lack of educational opportunities, may not have been recorded in the self-completion questionnaires as direct questions on these topics were not asked.

## Methods

All interviewees had completed ALSPAC Family History Questionnaires either online or on paper versions that were returned by post. The participants indicated on the questionnaire that they would be happy to be interviewed. Immediately prior to the interview the participants signed a consent form indicating that they had read and understood the Participant Information Sheet which states that “The results of the research will be written up and submitted as scientific papers and/or presented at academic meetings.” The participants were able to ask questions and clarify any concerns they might have about the Study before signing the consent form. The structured interviews (see extended data ( Birmingham *et al.*, 2021a)) and associated documents for participants (see extended data for: invitation letter, confirmation letter, information sheet, consent form, family trees, thank you letter, Helpline contact ( Birmingham *et al.*, 2021b)) were reviewed by the ALSPAC Ethics and Law Committee and granted a favourable opinion (Ref 63604 05/03/2018). This Committee reviews all ALSPAC primary data collections and is therefore familiar with all that is asked of the cohort ( Birmingham, 2018).

### Pilot

The Ethics Committee also approved a small pilot to ensure that the recording equipment, interview room and structured interview were fit for purpose. Six volunteers were invited to take part in the pilot study. These pilot participants were either current, previous or honorary members of ALSPAC staff and comprised of three female and three male individuals of approximately the same age range as the ALSPAC Study Mothers and Fathers. They were asked in person or by email if they would be happy to participate in the pilot before being sent the questionnaire (as they had not completed the ALSPAC Family History questionnaire which was to be validated by these interviews), Participant Information Sheet, Family Tree and covering letter. The consent form was signed immediately prior to the interview. A copy of the recording was offered to these volunteers and a brief report on the pilot study (see extended data ( Birmingham *et al.*, 2021c)) was sent to the six individuals. A few minor adjustments were made to the interview before the main study commenced. This included labelling by name the interviewee’s relatives on the family tree during the interview to help identify which relatives were being spoken about. Also, documenting dates on the family tree which enabled a rapid calculation of the relatives’ ages and thus if specific stresses or traumas took place during their childhood. These family trees were either taken away by the pilot volunteer or destroyed after the interview. It also became clear that to avoid repetition it was best to ask about the interviewees’ maternal (or paternal) grandparents first before asking about their mother (or father). One pilot interviewee advised that the recorded interviews should be encrypted in line with University policy before electronic transfer to the interviewees. This proved to be straightforward.

### Interviewee selection

All the interviewees had completed the ALSPAC Family History Questionnaire either online or on paper versions that were returned by post. In the questionnaire, they indicated that they would be happy to be contacted about a follow up interview. This was confirmed when they replied to a letter sent by ALSPAC staff with gave details of the interview and asked how they would prefer to be contacted (by email or by phone). Individuals were invited to be interviewed, but if they did not attend for whatever reason, another individual was selected, until we secured 100 interviews. The 100 interviewees were selected on gender and social economic status as defined by the Index of Multiple Deprivation (IMD) postcode classification ( UK Government 2019). They comprised 52 Study Mothers and 48 Study Fathers, just under half of whom had returned the questionnaires by post and the others had completed the questionnaire online. Although the selection process aimed to encompass a broad range of social economic status, half of the interviewees were in the three least deprived deciles, which reflects the social class of the ALSPAC cohort. The interviewees were contacted by the two ALSPAC interviewers and appointments made before being sent a confirmation letter, participant information sheet and family tree (see Birmingham *et al.*, 2001b). More Study Mothers than Fathers either did not reply, cancelled or did not attend (See Table 1).

**Table 1.** Interviewees’ Deprivation Scores.

	Study Mothers		Study Fathers		Final Total	
IMD decile	Interviewed	DNA	Interviewed	DNA	Interviewed	DNA
**10**	12	2	13	1	25	3
**9**	8	1	8	2	16	3
**8**	2	2	7	0	9	2
**7**	10	4	6	2	16	6
**6**	9	1	6	2	15	3
**5**	3	3	2	2	5	5
**4**	1	1	2	1	3	2
**3**	6	2	2	0	8	2
**2**	0	0	1	0	1	0
**1**	1	0	1	0	2	0
**TOTAL**	**52**	**16**	**48**	**10**	**100**	**26**

**IMD - UK Government 2019 Index of Multiple Deprivation from full postcode** **DNA - either did not attend, did not reply to invitation or cancelled**

The interviewees were born between 1944 and 1973 (aged between 46 years and 75 years when interviewed). Their parents (n=200) were born between 1897 and 1951 and their grandparents (n=400) between 1862 and 1923. All but 11 (5.5%) of the interviewees’ parents and 27 (6.75%) of their grandparents were born in the United Kingdom. Ten of the interviewees’ grandparents were born in the Republic of Ireland, the other relatives of interest were born as far afield as New Zealand, Australia, Singapore, India, South Africa, Canada, USA, Turkey, Austria, Italy, Germany and France.

A few interviewees were accompanied by their parent(s) (or parent-in-law), who provided first-hand accounts of their childhoods. This generation were especially pleased to recount their childhood experiences, as one said:

“… not many people had time to listen to their family histories these days.”

Many interviewees also brought documents and photos as aide-mémoires during the interview.

### Structured interview

Karen Birmingham, Research Fellow, and Yasmin Iles-Caven, Senior Research Associate, conducted the interviews. Both have extensive knowledge of ALSPAC having worked on the Study from the beginning in a variety of roles. Both trained at the British Library in conducting and recording Oral Histories and had conducted oral histories on key ALSPAC staff for other projects.

The ALSPAC interviewees were invited to attend the ALSPAC headquarters in Bristol. They were interviewed in a designated office within the ALSPAC clinic area which most were familiar with from previous visits to ALSPAC. Free parking was available to all interviewees. They were offered their travel expenses although many declined to be reimbursed. All interviewees were enthusiastic about ALSPAC as a whole and were eager to take part in the Family History interviews.

**Figure 1.**
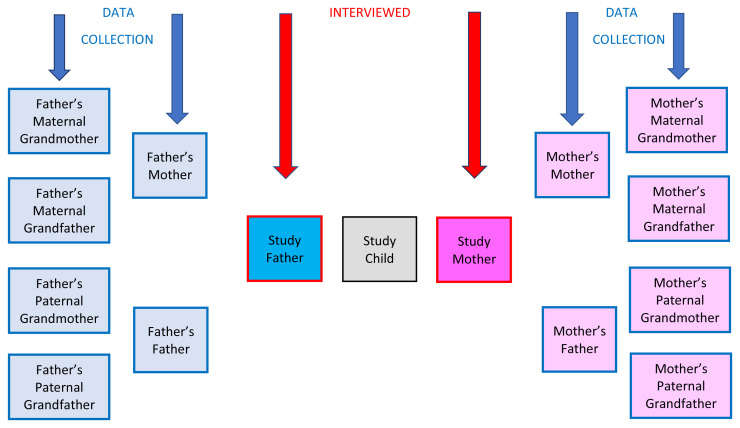
Ancestors of Interest.

Each interviewee was asked the same set of questions (see extended data: structured interview ( Birmingham *et al.*, 2021a)) about the childhoods of six relatives (see Figure 1). Childhood was defined as 16 years or under. The questions encompassed: i) date and place of birth and if the relative had moved during their childhood; ii) number and gender of the relative’s siblings and if older, younger or a twin; iii) age of onset of smoking and if mothers or grandmothers smoked when pregnant with, respectively, the interviewee or interviewee’s parent; iv) place, date and cause of death if the relative was no longer alive; v) information on 19 potentially traumatic situations in their childhoods such as serious illness, death or serious illness of a parent, being taken into care, not having enough to eat, being in a war situation or being subject to or witnessing violence. The ages at which the relative experienced the traumatic situations distinguished between ages <6, 6–11 and 12–16 years.

The duration of the interviews ranged from 25 minutes to 1 hour and 59 minutes. The interviews were audio recorded and the interviewers made notes throughout the interview. At the end of the interview, the interviewees were offered a £20 voucher as a token of gratitude for their time and effort and sent a copy of the recorded interview (encrypted) if they so wished. They were thanked, not only for agreeing to attend the interview but for all they had done over nearly three decades for the Study.

### Data entry

Immediately after the interviews, the interviewers entered the interview data into a REDCap data base (Version 9.1.0) ( Harris *et al.*, 2009; Harris *et al.*, 2019) for future validation against the self-completion questionnaires. Notes taken during the interview were entered also and it is from this text that quotations have been taken for this paper.

## Themes

### Morbidity and mortality within the families

Illness, both in the ancestors of interest but also in their family members, was often a source of severe stress, not only due to some deaths of parents or siblings but also as a consequence of isolation regimes, treatments or occasionally convalescence procedures.

**Figure 2.**
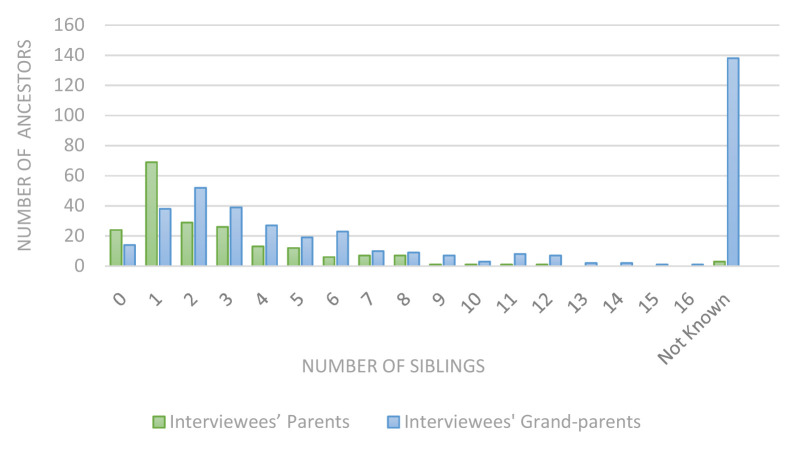
Ancestors’ siblings.

Perhaps unsurprisingly, there were several descriptions of exceptionally large families: three of the interviewees’ parents had 10 or more siblings, as did 24 of the interviewees’ grandparents. The largest of these families had 17 children (see Figure 2). Many anecdotes were recounted. One interviewee’s grandmother had 12 children plus two miscarriages:

“She buried the foetuses at a sacred ancient site nearby, where she knew they would not be disturbed.”

Many of the siblings did not survive to adulthood. Quite often the age and cause of death was unknown and especially with the grandparents, often uncertainty was expressed about the numbers, ages and gender of the siblings also.

Several deaths in childbirth of mothers as well as siblings were reported. One interviewee’s grandmother, aged 5, lost her mother who died in childbirth delivering twins who also died:

“The twins were buried in the coffin with their mother. The coffin had to be made very wide to accommodate them, each under the mother's arms, and a window had to be taken out in order to get the coffin in and out of the house.”

There was one distressing account of a relative’s sibling’s death. The participant’s mother had witnessed her own mother suffocate her new-born sibling as she could not cope with yet another child:

“It was probably thought to be a cot death but might not have even been registered.”

There was also an uplifting account of one mother:

“She was awarded a certificate from the local mayor for raising a child unlikely to survive.”

***Disease.*** Some specific diseases affecting the families were mentioned frequently: pneumonia, tuberculosis, scarlet fever, rheumatic fever and septicaemia. Other diseases were mentioned less frequently: asthma, whooping cough, rickets, goitre, gastroenteritis, meningitis, diphtheria, typhoid, poliomyelitis and Spanish flu. Causes of death in the families included: ruptured appendix, stomach ulcer; heart attack; pernicious anaemia (after being gassed in World War I); gangrene; lung cancer; chronic nephritis.

***Accidents.*** Accidents were often cited as the cause of deaths within families. Some seemed common (e.g., falls and road traffic accidents), others less prevalent (e.g. train accidents) and others somewhat unusual (e.g. sexual game). One interview recounted their relative’s siblings’ deaths:

“… one of 14 children, all survived childhood but one of the younger siblings died youngish in a motor bike accident and one died accidently (impaled on something).”

Another interviewee’s mother’s 2-year-old younger brother died:

“… four days after pulling a cauldron of boiling hot liquid onto himself.”

Yet another interviewee’s grandmother’s brother:

“… drowned in a well in the garden.”

One recounted an accident that was historically unique:

“He was on board the docked HMS ( *British warship*); some crew were on board and some on land and there were families visiting (cinema on board). There was a catastrophic explosion due to incompetent storage of the explosives; they were running the ship as if it was an old wooden ship, 'block heads' [doors between parts of the ship] had been left open so fire ripped through ship leaving hundreds of crew and visiting families dead. He was classed as missing. There was some cover up of incident”.

***Hospitalisation, isolation and other treatments***. The interviewees’ relatives who survived the diseases prevalent at the time often had to endure many months as young children in hospital, in isolation, unable to see their parents except occasionally through glass. One interviewee’s mother aged 6, contracted scarlet fever and was moved into isolation in hospital for 14 weeks:

“She didn't see her mother for 10 weeks and didn't understand why she was there and if she would ever go home. The food was awful, the same every day and she was physically neglected. She didn't have her hair washed until her mother visited her after 10 weeks.”

Although hospitalisation was not always a traumatic experience, for some it certainly was, due to neglect or absence of parental visits frequently due to difficulties in travelling to the hospital. One interviewee’s grandmother carried her 4-year-old daughter, who was suffering from diphtheria, three miles to hospital. Another interviewee’s father at the age of 6 was admitted to hospital with pneumonia, which was traumatic enough, but what was considered even worse was a two-month post hospital trip to Switzerland alone to recuperate:

“It was a horrible experience; he was locked in a dark cupboard for hours on end. He had brand new clothes when he was sent there but when he came back, he was in old second-hand clothes.”

Other interviewees described the trauma of their relatives’ treatments. One interviewee’s mother at the age of 6 or 7 had severe pneumonia:

“She was left with a 12-inch square scar on her back from hot poultices used to treat her.”

Another interviewee’s grandmother had her appendix removed on the kitchen table as a child. Yet another aged between 6–11 years had ulcers behind her eyes and after treatment had to spend hours in a dark cupboard to aid recovery. More positively, there were two examples of penicillin being successfully trialled on interviewees’ mothers: one for septicaemia following an infected cut to her knee, the other following surgery (pulmonary lobectomy) after developing pleurisy.

***Mental health.*** The interviewees frequently described mental health problems from which their relatives suffered, often exacerbated by extreme poverty or experiences during the War(s), which would almost certainly be classified these days as post-traumatic stress disorder (PTSD). The accounts included numerous mentions of alcoholism (frequently with accompanying violence) and several suicides, although some conditions were less well defined:

“Alcoholic stepmother did not like housework so *(interviewee's mother)* had to do it. She did her own laundry from about 11. Friends not allowed into house and had to wait outside. Frequently kept home from school in order to collect firewood or cigarette ends which her stepmother rolled into cigarettes. *(Interviewee's mother)* found this degrading but it stopped stepmother from shouting.”
“Her mother had 'a temper'; when she was an older teenager, her mother ran down the street after her with a pan of boiling fat. Her mother used to disappear for days at a time. It was said that her temper was much worse during the full moon.”
“His mother died of TB. Father called up. Until 1945/6 was looked after by nuns (Franciscan convent). Was never spoken to by nuns. Consequently, difficulty in relating to others and lack of social skills.”
“He was captured by the Black and Tans and imprisoned in terrible conditions for a year. They lived in little huts with one tap, few bedclothes and were terrified; he never recovered.”

## Effects of World Wars

**Figure 3.**
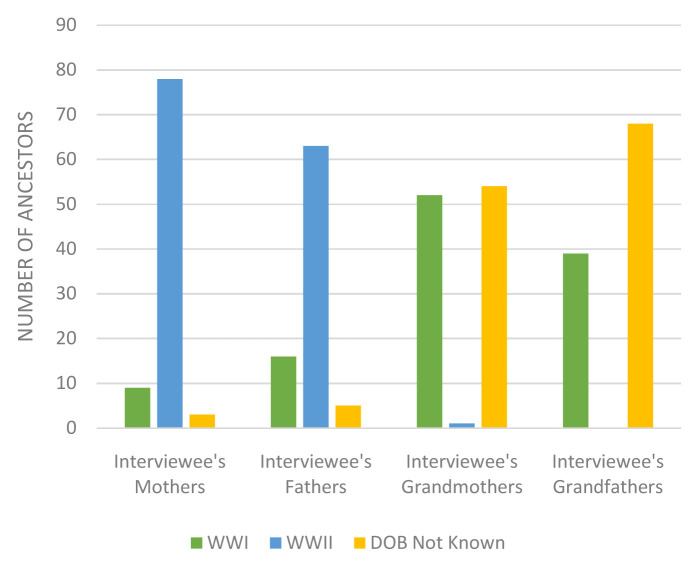
Ancestors 0–16 years old during World Wars.

Many of the interviewees’ ancestors were children (0–16 years) during the World Wars although there were a considerable number of interviewees who did not know the date of birth of their ancestors, especially their grandparents (see Figure 3). It is likely that many of these grandparents with an unknown age were also children during World War I.

### World War II

World War II was not necessarily traumatic for those who were children during the six years of war. Several interviewees expressed similar opinions, in that that their relatives:

“Didn't think of World War II as traumatic, just got on with it.”
“War was not traumatic but exciting, an adventure.”
“They used to collect shrapnel and painted curb stones white (for safety during the blackout).”

For others, the impact of the War was profound. For many their own fathers were away for many years, as were their siblings or other relatives:

“An uncle ended up in Buchenwald concentration camp, missing in action having been shot down, which worried the whole family. He went with brown, came back with grey hair.”
“Brother died at 16 years - torpedoed in the War.”
“…was himself in the Navy during WWII on the Atlantic convoy. Was a gunner on a DEMS ship (Defensively Equipped Merchant Ship - i.e. a merchant ship with Navy installed guns). He was torpedoed in 1942 and spent several days on a raft (oil in water perforated his ear drum). Suffered terrible nightmares after the war (screaming in the night).”

One interviewee’s grandmother was living in Europe during World War II:

“She had a very hard life during World War II and for many years afterwards. Her husband was randomly shot dead by Russian soldiers while walking home in the last week of the war. Russian soldiers were then billeted in her house for nine years after the war. Could have been worse as she was not raped as many were.”

Many, of course, never returned.

### Evacuation

Some interviewees’ parents were evacuated during World War II, which proved to be a positive experience for some, although for others it was thoroughly traumatic:

“Her father was absent for 12 years during her childhood almost certainly in prison. He was an extremely violent alcoholic: once tried to throw one of his children from the window of a high rise flat. Her mother moved around the country a lot. Her elder brother (by 12 years) remembers having to put mother to bed 'under a bush' at times. She was put in care with 2 younger sisters (not known at what age) but was back with her mother between 7–9 years. Her mother was unpredictable and violent: she threw a member of care home staff down the stairs when she discovered that her daughters had head-lice. During the war, she was evacuated (alone, not with her siblings) to Devon where she lived with a single woman and her cocker spaniel; this was a happy period of respite from her family, living for several years by the coast. Her mother insisted she returned aged 14 so she could go out to work. She got a job in a clothes factory, but all her wages were taken by her mother.”

One maternal grandmother attended the interview and gave a first-hand account of her evacuation:

“The school was responsible for moving all the children en masse to [ *small country town*] and the evacuees were chosen by local people. I was the penultimate child to be picked, along with the most unpopular child. I felt unwanted and was unwell from the train ride.”

Other families had evacuees housed with them:

“The family took in evacuees during World War II - often 6 in a small 3-bedroom house.”
“He had to share his bed with evacuees; one boy from Surrey and then one from Folkestone.”

Although bed-sharing was also the result of poverty:

“War did not have a big impact on him. There was enough food as a child but little space (top-to-tail in bed).”

### World War I

Of the 285 interviewees' grandparents whose year of birth were known, just over half were children during World War I, five were born after the war, and 133 were adults when the war broke out. Their war experiences frequently affected their (future) children. Some of the grandfathers lied about their age in order to join up:

“Grandfather and his two brothers lied about their age and enlisted in ‘The Glosters’ as young as 15. Brother 1: Survived the war but whilst waiting for a train home was playing with a grenade which exploded and killed him. Brother 2: Died on the Western Front. Grandfather was wounded in battle and was left for dead in No Man's Land. German soldiers were dispatching the British injured, but the two that found grandfather, had a bet on if he would live or die from his wounds. He was taken to a hospital, survived and spent the rest of World War I in a POW *(Prisoner of War)* camp.”

Others were in protected professions that exempted them from service. Two of the interviewees’ grandfathers were conscientious objectors during World War I:

“Following his arrest and a military tribunal, he was interned - nine months hard labour, commuted to 140 days. He was categorised as 'Class A' - a genuine objector. He was then employed as a road mender for the duration of the war under the ‘Home Office Scheme’. These ‘schemes’ were work camps set up by the government in 1916 after it was decided that 4,378 prisoners were 'genuine' objectors. Some camps were relatively comfortable, others barely habitable. Work varied from the unpleasant (making fertiliser from dead animals) to the utterly futile (manual labour for non-existent projects).”

For those who served, there were many descriptions of the long-lasting physical and mental effects and the stresses endured by their children and wider families:

“Her mother's sister had seven sons, all lost in World War I.”
“… father had been gassed in World War I, coped but was never psychologically robust thereafter.”
“Aunt said he was a nice boy when he went but far from nice when he returned.”
“Registered as dead in WWI but was rescued from a pile of dead bodies. Bullet ricocheted off his head when he agreed to cross 'machine gun alley' if his friend, who was a tailor, agreed to make him a suit for his wedding (which he did). Became mute for some time after the war. On wedding day, clap of thunder again turned him mute for several weeks.”
“Went to war in France before he was 18. Never spoke about it. Awarded a medal but never took it out of the postal packet in which it was sent to him.”

Occasionally there was a positive outcome: one interviewee’s grandfather:

“… was one of seven brothers all called up and all survived the war.”

Another interviewee recounted that:

“Her mother’s brother died during World War I; she married the man who came to break this news to her.”

## Poverty

Over 20 of the interviewees described at least one, if not more, of their relatives as being brought up in financially stricken households or even in “grinding poverty”:

“Ancestors were all cotton mill workers - very poor with two families sharing a one-up-one-down house.”
“One of 12 siblings in a tiny cottage. Father was a chimney sweep.”
“Family quite poor - had to make shoes out of cardboard.”
“Frequently not enough to eat, would be sent out to catch rabbits for the pot. Raised money for shoes by carol singing.”

Lack of food was a recurring theme for many of the interviewees’ relatives:

“Not enough to eat throughout childhood; brother had to pick up 'two bob' once a week from a generous neighbour as he was another mouth to feed.”

Numerous families grew their own vegetables, kept chickens or other livestock or supplemented their diet with rabbits or game:

“During the war, he and his mother and all his siblings moved to live with an aunt in the Somerset countryside. Was not enough to eat; one time he went to feed his pet rabbit after supper, only to discover they had just eaten it.”
“They never knew where their next meal was coming from. An elderly woman in the village used to make a pudding every Wednesday which one of the kids had to collect. Mother boiled sheep's head for a meal and used the stock for stew the next day.”
“Lived on a very small farm with chickens and caught perch from the local river.”

### Children cared for by others

Several of the interviewees’ relatives were not brought up by their parents, either due to their parent’s death or illness or due to poverty. A few were in and out of the workhouse during childhood, one was brought up in Barnardo’s (children’s home provided by the charitable organisation), others were taken in by wealthier associates, some of whom were related but some were not:

“Aged 13 his mother died in the workhouse from septicaemia after an abortion. He was looked after by his father but was in and out of workhouse until taken in by his maternal grandmother.”
“Aged 13 grandmother's mother died, cause of death not known. Then in World War I grandmother's father killed. The eldest daughter cared for the kids as best she could. Local vicar was given money to support them, but he drank it away. Two siblings sent to Barnardo’s, the rest farmed out to family.”
“After parents' deaths in 1920, the eldest daughter married her boyfriend so that they could legally become guardians of the younger children, with some support from the wider family.”
“Brought up by aunt and grandmother in ( *Scottish town*). They doted on him; spoilt rotten. His mother was 19 when he was born and possibly not married. She offered to have him back when he was 9. He got to the station but decided he did not want to go and live with her. Aunt and grandmother delighted with this decision.”
“Aged 9–10, taken in by a local relatively wealthy doctor because it would give her a better chance. Created distance between her and her family.”
“Impoverished family but from early teenager moved to live with rich relatives (live in servant). This was a genteel household and caused some division in the family as she became a snob as she went up in the world.”
“Orphaned at 14. Two sisters aged 13 and 15 went into Service. Two younger brothers and a younger sister went to an orphanage. He worked hard and eventually managed to bring the siblings home.”

## Violence

Almost certainly poverty and other stresses increased the incidence of alcoholism and with it domestic violence, although some violence was considered normal and affected rich and poor alike:

“Children were spanked or strapped but not considered violent, very much what happened at the time.”
“Beatings by teachers and prefects was a way of life, not considered traumatic, and children learnt to keep out of trouble.”
“Given a good hiding when looking after his sister and she lost her hat in a river. Thought to be quite normal for that era.”

Other accounts went far beyond what could or should be described as normal:

“At school used to get a thrashing if Irish was spoken.”
  “Schools were horribly violent; if children didn't understand something they would be physically punished.”
“He had a 'strap' which came out for any minor misdemeanour, e.g., being late home from milk round.”
“Smoking nipped in bud at early age when made to smoke whole packet and beaten but took snuff all his life.”
“Miserable at school - subjected to much violence, bullying and almost certainly sexual abuse but happy times in holidays when he stayed with his cousins.”
“He had two brothers, and their father was a strict Victorian clergyman who beat them severely and regularly when they were young to ‘beat the sin out of them.’”

One interviewee described harrowing, criminal violence:

“Abuse and violence as a child turned him into a very violent and abusive adult who consistently raped the eldest daughter as a child until she was diagnosed with *(a severe chronic illness)* and then frequently raped youngest of her sisters. He also sexually abused one of his grandchildren. The eldest daughter describes her childhood as very mixed as he was also very loving and kind.”

## Education

Many of the interviewees’ grandparents had to leave school in their early teens or even younger to start work; at least 20 of the interviewees mentioned one or more relatives going into ‘service’ (one at the age of 11) and 17 interviewees had at least one relative serving apprenticeships, usually from the age of 14 but some from as young as 12. Two of the grandparents set out, aged 14, on their own, to start a new life in Australia, though both returned to the UK as adults. One interviewee’s grandparent was known to be illiterate as well as many with hardly any education:

“Her mother died when she was about 9. Had to stop school and stay at home and look after her father and brothers - life of drudgery at that point.”
“Before the age of 6, he moved to Ireland to be brought up by his aunt as there were 'too many in the family'. Moved back to Bristol when he was old enough to go to work. Had one year of schooling when back in Bristol but no schooling at all in Ireland - just used to roam around the countryside.”

A few relatives attended fee paying (public or independent) schools and there were some highly successful relatives:

“Qualified from Manchester Technical College as an engineer; received an OBE, CBE and Knighthood for services to Engineering.”
“Attended Hornsey Art School, became famous artist/illustrator. Had a privileged background. He suffered a downturn in income in World War II with a lack of commissions for book illustrations”.

## Discussion

The childhood stresses of the ancestors of the ALSPAC parents, as detailed in the self-completion Family History Questionnaires, will be extensively analysed with particular attention to possible epigenetic consequences in their descendants. Meanwhile, the interviews provide insightful descriptions of the types of stresses endured in childhood by the cohort’s ancestors. As far as we are aware, no other longitudinal cohorts have conducted similar investigations into participants’ ancestors. Much of this anecdotal information will have been missed in the self-completion questionnaires as lack of educational opportunities and siblings’ deaths were not specifically asked about. Similarly, reports of close relatives killed or reported missing in action during war time had profound effects on the parents/grandparents and were only reported at interview.

There were some interviewees that knew absolutely nothing about some relatives for various reasons, for example: early death of that person and so they had never met, or only knew them at a very early age; illegitimacy; adoption or falling out with the rest of the family due to their or their parents’ marriage. Some veterans of the world wars had been so traumatised by their experiences that they never spoke in detail about them, but the interviewee could report this.

The interviews were mainly focussed on stresses or traumas in childhood so accounts and anecdotes naturally give more negative impressions of the childhoods than might otherwise be the case, but many interviewees also described their relatives having secure, stable childhoods. There were some descriptions of wealthy relatives, with nannies and governesses and expensive boarding schools, but by far the more frequent accounts were of poverty, often severe, with related lack of education, illiteracy, alcoholism and violence, alongside the devastating effects of the World Wars. Amidst the disturbing accounts were some amusing and curious anecdotes:

“When he was told that his fifth child was another daughter (four daughters previously) he was known to say 'sod it, sod it, sod it' and then spoilt that daughter ever afterwards as he felt so guilty. (Sixth child was a boy.)”
“When he married, his non-Jewish wife, he was disowned by the rest of the family who considered him dead and conducted his fake funeral.”
“…as the 7 ^th^ son of a 7 ^th^ son it was thought he could commune with nature.”
“After the War, moved back to the rectory although her husband (the rector) had died. Allowed to rent out rooms including the chicken coop to Ted Hughes” (later to become poet laureate).

Many children were brought up, not by their parents but by relatives and/or left home very young to ‘go into service’ or start work, including the two grandfathers who went to Australia alone, aged 14, to begin their lives working on ranches in the outback. Perhaps the predominant impression of these many struggling families, is their remarkable resilience; all went on to have children or grandchildren who are stable enough to participate for nearly three decades, entirely altruistically, in contributing to the intensive data collection required of ALSPAC participants.

## Data availability

### Underlying data

ALSPAC data access is through a system of managed open access. The steps below highlight how to apply for access to the data included in this paper and all other ALSPAC data:

1. Please read the ALSPAC access policy which describes the process of accessing the data and samples in detail, and outlines the costs associated with doing so.

2. You may also find it useful to browse our fully searchable research proposal database which lists all research projects that have been approved since April 2011.

3. Please submit your research proposal for consideration by the ALSPAC Executive Committee. You will receive a response within 10 working days to advise you whether your proposal has been approved.

The Study website contains details of all the data that is available through a fully searchable data dictionary and variable search tool ( http://www.bristol.ac.uk/alspac/researchers/our-data/).

### Extended data

Figshare: ALSPAC Family History Structured Interview. https://doi.org/10.6084/m9.figshare.13951580.v1 ( Birmingham *et al.*, 2021a).

This project contains the following extended data:

- Interview Qu for Mothers.docx
- Interview Qu for Fathers.docx

Figshare: ALSPAC Documents for Family History Interview Participants. https://doi.org/10.6084/m9.figshare.13951799.v1 ( Birmingham *et al.*, 2021b).

This project contains the following extended data:

1 Family History Validation Interviews_Invite Letter and Reply Slip.docx
2 Family History Validation Interviews PIS.docx
3 Family History Validation Interviews Confirmation Letter and Employers Letter.doc
4 Family History Validation Interviews_Family Tree Mothers.docx
5 Family History Validation Interviews_Family Tree Fathers.docx
6 Family History Validation Interviews_Consent Form.docx
7 Family History Validation Interview Thank you Letter.docx
8 Cruse helpline and website.docx

Figshare: ALSPAC Report on Family History Interview Pilot Study. https://doi.org/10.6084/m9.figshare.13951949.v1 ( Birmingham *et al.*, 2021c).

Extended data are available under the terms of the Creative Commons Attribution 4.0 International license (CC-BY 4.0).
