# Supplementary material for: ALSPAC parents’ descriptions of childhood stresses in their parents and grandparents
Source: Wellcome Open Res. 2022 Jun 14;6:115. Originally published 2021 May 14. [Version 2] doi: 10.12688/wellcomeopenres.16732.2 (PMC9156897; doi:10.12688/wellcomeopenres.16732.2)
Supplement: Peer review history

# Peer review: Reviewer response for version 2

**DOI:** 10.21956/wellcomeopenres.19919.r51053

**Published:** 2022-06-14

**Authors:** Alicia Matijasevich

**Affiliations:** 1 Department of Preventive Medicine, Faculty of Medicine FMUSP, Universidade de São Paulo, São Paulo, Brazil

Everything is ok for me.

Is the work clearly and accurately presented and does it cite the current literature?

Yes

If applicable, is the statistical analysis and its interpretation appropriate?

Not applicable

Are all the source data underlying the results available to ensure full reproducibility?

Yes

Is the study design appropriate and is the work technically sound?

Yes

Are the conclusions drawn adequately supported by the results?

Yes

Are sufficient details of methods and analysis provided to allow replication by others?

Yes

Reviewer Expertise:

Epidemiology, Maternal and child health, cohort studies

I confirm that I have read this submission and believe that I have an appropriate level of expertise to confirm that it is of an acceptable scientific standard.

# Peer review: Reviewer response for version 1

**DOI:** 10.21956/wellcomeopenres.18453.r50583

**Published:** 2022-05-31

**Authors:** Barry J. Milne

**Affiliations:** 1 Centre of Methods and Policy Application in the Social Sciences (COMPASS), University of Auckland, Auckland, New Zealand

This study reports the results of qualitative interviews with a sample (n=100) of parents from the Avon Longitudinal Study of Parents and Children (ALSPAC) study.  The interviews focused on the parents and grandparents of the interviewees (grandparents and great-grandparents of the ALSPAC children), and the trauma and stresses that the parents and grandparents experienced in their childhood.  Several types of trauma were commonly reported, such as illness and death, family separation, violence, alcoholism, poverty, food insecurity, and the impact of the world wars. The authors conclude that despite the struggles reported and the necessary focus on negative experiences, remarkable resilience was shown by the parents and grandparents of interviewees, who managed to thrive (to some extent) and produce children and grandchildren now involved in ALSPAC.

 This is fascinating research, which reminds us again of the widespread difficulties faced by recent generations.  Thank you to the authors and especially to the ALSPAC interviewed parents for providing these insights.

 I have no major criticisms of the paper, just a few suggestions to help clarity.

1. The methods in the abstract could be rewritten to emphasize that the study reports themes relating to stress and trauma in previous generations from interviews with the parents.  As written, the methods seem to indicate that results from interviews with n~7000 parents will be discussed, along with validation using interviews from n=100 parents.  Neither is the case.  While the n=100 might be known as the ‘validation sample’, nothing relating to validation is reported.  Results from interviews with parents about their parents and grandparents are reported, and this should be stated in the methods.
2. I was a little confused about how many interviews were actually conducted.  Table 1 indicates it might be n=74, as 26 did not attend.  Is this the case?  If so it should be stated in the text under interviewee selection and also in the abstract.
3.  A related point is that it would be useful to indicate the number of parents and grandparents of interviewees that were discussed in the interviews.  Is it n=148 and n=296, i.e., 74x2 and 74x4?  This would help with the interpretation of some reported counts, e.g., “All but 11 of the interviewees’ parents and 27 of their grandparents were born in the United Kingdom”.  11 of how many and 27 of how many?  Percentages here would be helpful too, and perhaps also in Figure 2.  Figure 3 I think is fine with just counts reported.
4. The paper indicates that there are some things that the interviewees did not know about the parents and particularly their grandparents (e.g., years of birth, number of children).  Did the researchers get a sense from the interviews if interviewees sometimes did not seem to know enough to report about the trauma of their parents and grandparents?  Or did researchers get a sense that trauma was seldom discussed in some families?  If so to either, perhaps this could be discussed in the paper.

Is the work clearly and accurately presented and does it cite the current literature?

Yes

If applicable, is the statistical analysis and its interpretation appropriate?

Not applicable

Are all the source data underlying the results available to ensure full reproducibility?

Yes

Is the study design appropriate and is the work technically sound?

Yes

Are the conclusions drawn adequately supported by the results?

Yes

Are sufficient details of methods and analysis provided to allow replication by others?

Yes

Reviewer Expertise:

Life-course epidemiology; family history research

I confirm that I have read this submission and believe that I have an appropriate level of expertise to confirm that it is of an acceptable scientific standard.

# Response

**Published:** 2022-06-08

**Authors:** Yasmin Iles-Caven

**Affiliations:** University of Bristol, UK

Thank you for your review of our paper.  We have made alterations as follows to the text:

**Comment 1.**  We have clarified the Methods within the Abstract.

**Comment 2.**  Table 1. Have labelled the R-H column 'Final Total'.  In total 100 interviews were undertaken, and we have clarified this in the Methods/Interviewee selection.

**Comment 3**. Methods/Interviewee selection: Have added percentages and total numbers of ancestors.

**Comment 4**.  Additional text has been put in the Discussion concerning the fact that some interviewees knew absolutely nothing about some ancestors and examples of the reasons for this.  It is possible that some families did not talk much, if at all, of some traumas and therefore the interviewee would not have known about it.  Many interviewees confessed to talking to family members in advance of the interview to obtain more information. However, we, the interviewers, never felt that any interviewee 'held back' on details.

# Peer review: Reviewer response for version 1

**DOI:** 10.21956/wellcomeopenres.18453.r43898

**Published:** 2021-05-24

**Authors:** Alicia Matijasevich

**Affiliations:** 1 Department of Preventive Medicine, Faculty of Medicine FMUSP, Universidade de São Paulo, São Paulo, Brazil

I would like to thank you for the opportunity to review this very nice manuscript. It presents interesting results of transgenerational trauma/stress, a topic that has wide interest and relevance. It is very well-written, and structured. Its results are presented in a clear and didactic way. The article describes situations, some of them very sad and extreme, that touched the reviewer. I imagine that remembering and sharing these experiences was a moving experience for both interviewees and interviewers. Just as a minor suggestion, I think that the discussion could be expanded a bit including the following information:

1. Do the authors know if other existing cohorts have conducted investigations similar to the present study? If so, could some of the results be compared?
2. It is written: “…the validation interviews provide insightful descriptions for the types of stresses endured in childhood…” Please be more specific regarding how the present results may be incorporated into future work on this subject.

Is the work clearly and accurately presented and does it cite the current literature?

Yes

If applicable, is the statistical analysis and its interpretation appropriate?

Not applicable

Are all the source data underlying the results available to ensure full reproducibility?

Yes

Is the study design appropriate and is the work technically sound?

Yes

Are the conclusions drawn adequately supported by the results?

Yes

Are sufficient details of methods and analysis provided to allow replication by others?

Yes

Reviewer Expertise:

Epidemiology, Maternal and child health, cohort studies

I confirm that I have read this submission and believe that I have an appropriate level of expertise to confirm that it is of an acceptable scientific standard.

# Response

**Published:** 2021-06-04

**Authors:** Karen Birmingham

**Affiliations:** University of Bristol, UK

Thank you for this generous review.

 You ask if other cohorts have conducted similar investigations. To our knowledge no other cohorts have conducted similar interviews.

 You also ask how the present results may be incorporated into future work. The interviews were designed to validate the ALSPAC Family History self-completion questionnaires and will be analysed for that purpose. They provide historical context and vivid illustrations of the stresses endured by these generations, hence this brief descriptive paper. Professor Jean Golding continues to work on transgenerational epigenetics within the ALSPAC cohort.

# Response

**Published:** 2022-06-08

**Authors:** Yasmin Iles-Caven

**Affiliations:** University of Bristol, UK

Thank you for your review of our paper.  To add to Karen Birmingham's responses, we have put some additional text mainly in the discussion to clarify your points.
